# The new Editorial Board

**DOI:** 10.1007/s12471-016-0926-3

**Published:** 2016-12-08

**Authors:** J. J. Piek

**Affiliations:** AMC Heart Center, Academic Medical Center, University of Amsterdam, Amsterdam, The Netherlands

In this first issue of the Netherlands Heart Journal in 2017, I would like to introduce to you a new editorial board, consisting of an editor-in-chief and five deputy editors. It is an Amsterdam-based editorial board to facilitate close interaction between the board members. It is the ambition of the new editorial board to further improve the journal’s current impact factor as an index of its quality and success. The attractiveness of the journal for authors may be enhanced by offering a rapid review system, which requires close collaboration between the associate editors and the national editorial and international advisory board. Moreover, the Netherlands Heart Journal is an open access, peer review journal offering services without payment for authors in contrast with numerous other open access journals.

**Fig. 1:**
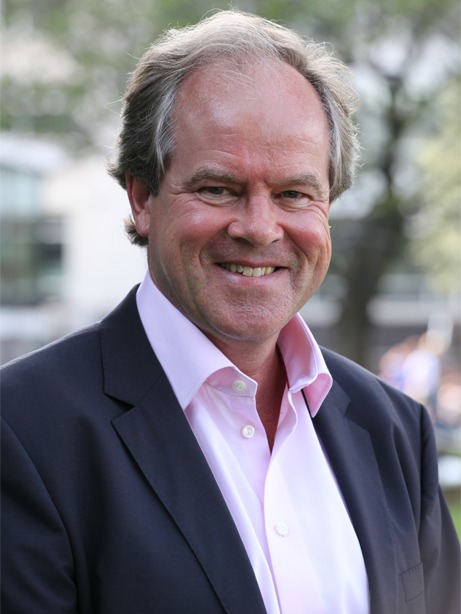
Jan J. Piek, PhD, Professor of Clinical Cardiology

Professor Jan J. Piek (Fig. 1) completed his residency program in cardiology at the Academic Medical Center of the University of Amsterdam (1984–1989), followed by a subspecialisation in interventional cardiology. He finished his thesis on the coronary collateral circulation in 1992. He was appointed as Professor of Clinical Cardiology in 1999. He has been co-chairman of the Department of Cardiology since 2004 and he was appointed director of the AMC Heart Center in 2008. The interests of the research group of Professor Piek include coronary haemodynamics, the inflammatory response in acute myocardial infarction and atherosclerosis, and the formation of coronary collateral circulation.

**Fig. 2:**
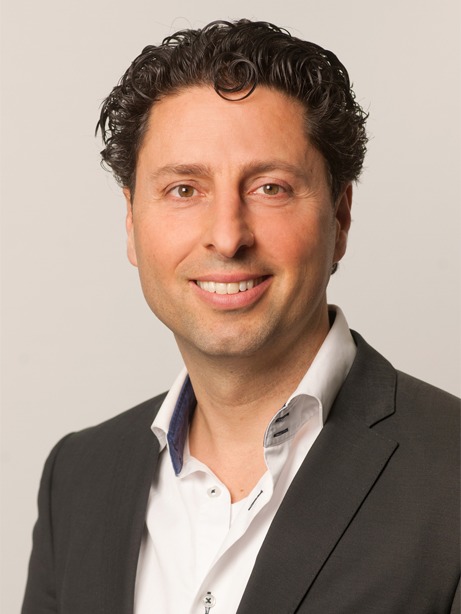
Yigal Pinto, MD, Professor of Cardiology

Yigal Pinto (Fig. 2) was trained as a cardiologist in Groningen in the Netherlands (1995–2001), and worked as a staff cardiologist at the University Hospital Maastricht (2001–2008) before moving to the AMC. He received basic research training in Boston (in the group of Victor J. Dzau) and in Berlin (in the group of Martin Paul).

The main research interest of the Pinto group (AMC, Amsterdam) is to identify novel mechanisms that can help treat or diagnose cardiomyopathies, such as genetic forms of DCM. His group focuses on RNA biology in relation to genetic variants that are related to heart failure and translates findings to biomarkers or therapeutic targets.

**Fig. 3:**
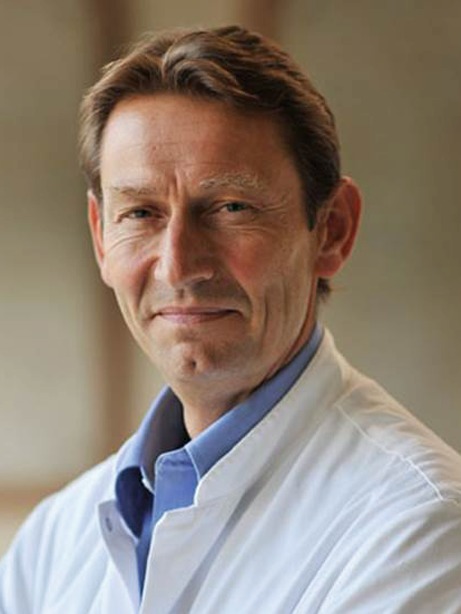
Robbert J de Winter, MD, Professor of Clinical Cardiology

Robbert de Winter (Fig. 3) studied biophysics in L﻿e﻿i﻿d﻿e﻿n (1976–1980, MSc: 1987) and medicine in Amsterdam (1980–1988). He was trained as a cardiologist (1988–1994) and interventional cardiologist (1994–1995) in Amsterdam. He has been a staff member of the AMC since 1994 and Professor of Clinical Cardiology since 2008.

The main research interest of his research group relates to acute coronary syndromes, diagnostics, risk prediction and outcome in STEMI and NSTEMI patients and biomarker studies. In addition, there is increased involvement in structural and congenital heart disease both in percutaneous procedures and in research projects.

**Fig. 4:**
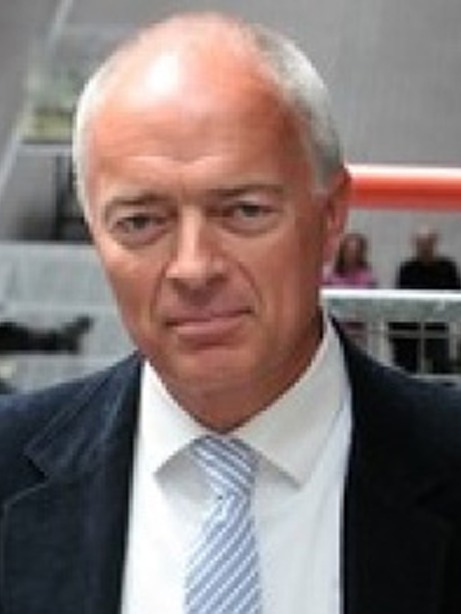
Ron Peters, MD, Professor of Cardiology

Ron Peters (Fig. 4) had all of his training at the University of Amsterdam: medical school, specialty certification in internal medicine and cardiology, PhD studies on intracoronary ultrasound imaging, and his appointment as Professor of Clinical Cardiology. His research topic is prevention of coronary artery disease.

**Fig. 5:**
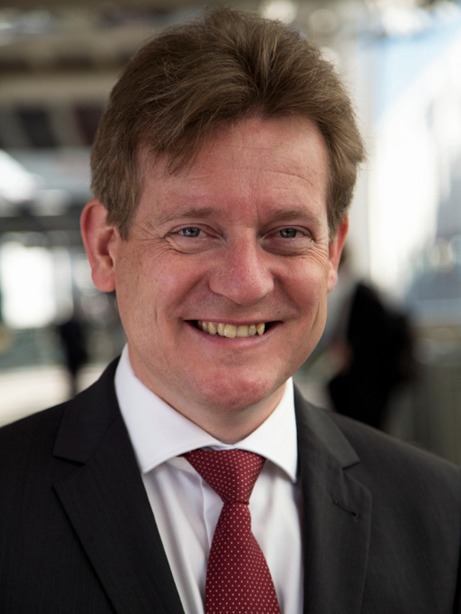
Joris R. de Groot, MD, PhD, Staff Cardiologist-Electrophysiologist, Head of Clinical Electrophysiology

Joris de Groot (Fig. 5) was born and raised in Amsterdam, the Netherlands. He studied medicine at the University of Amsterdam (MD 1997, *cum laude*), and defended his PhD thesis at that same institution in 2001. He was a research fellow with Michael R. Rosen MD (Columbia University, New York, NY) and with José Jalife (SUNY Upstate Medical University, Syracuse, NY). After his training as a cardiologist (2008) he subspecialised as a clinical electrophysiologist (2010), both at the AMC. The research interests of his group consist of mechanisms and treatment of atrial fibrillation, closely intertwined with the thoracoscopic atrial fibrillation ablation program and the Laboratory of Experimental Cardiology. Furthermore, his group publishes on arrhythmias in adult congenital heart disease.

**Fig. 6:**
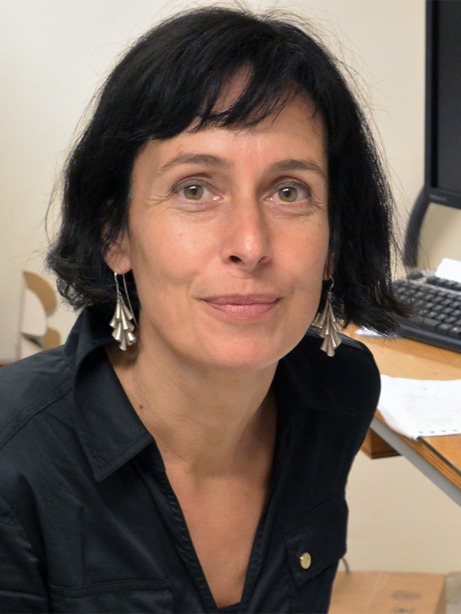
Jolanda van der Velden, PhD, Professor of Physiology

Jolanda van der Velden (Fig. 6) studied medical biology at the University of Leiden, and received a PhD in Physiology (1998) at the VU University in Amsterdam. In 2013 she became Professor of the Netherlands Heart Institute (Utrecht), and since 2014 she is chair of the Department of Physiology at the VU University Medical Center in Amsterdam. The main research interest of the Van der Velden group is to understand the role of cellular proteins in cardiac muscle cell dysfunction during the development of heart failure. In vitro studies at the cellular level are combined with in vivo imaging studies of cardiac function in patients. Currently, novel compounds are being tested in cardiac tissue systems and animal models aimed to prevent cardiac dysfunction.

The editorial board would like to take the opportunity to thank the previous board members and in particular Professor E.E. van der Wall, who served as editor-in-chief for almost three decades, initially as the editor-in-chief of the Nederlands Tijdschrift voor Cardiologie followed by the journal Cardiologie and finally the Netherlands Heart Journal. These efforts led to the current position of the Netherlands Heart Journal among the top of the 60 national society journals of the ESC-related countries. The editorial board acknowledges the support of the Netherlands Society of Cardiology in its endeavour to further improve the quality of the Netherlands Heart Journal.

